# Reconstruction with the right gastroepiploic vein during pancreaticoduodenectomy and total pancreatectomy to prevent left-sided portal hypertension: a report of two cases

**DOI:** 10.1186/s40792-023-01773-x

**Published:** 2023-11-20

**Authors:** Sanshiro Hatai, Keizo Kaku, Shinsuke Kubo, Yu Sato, Hiroshi Noguchi, Yasuhiro Okabe, Naoki Ikenaga, Kohei Nakata, Masafumi Nakamura

**Affiliations:** Department of Surgery and Oncology, Graduate School of Medical Sciences, Kyushu University, 3-1-1 Maidashi, Higashi-Ku, Fukuoka, 812-8582 Japan

**Keywords:** Pancreaticoduodenectomy, Total pancreatectomy, Portal vein, Left-sided portal hypertension, Gastric venous congestion, Splenic vein, Right gastroepiploic vein, Pancreatic cancer

## Abstract

**Background:**

Left-sided portal hypertension including gastric venous congestion may be caused by ligating the splenic vein during pancreaticoduodenectomy with portal vein resection or total pancreatectomy. The usefulness of reconstruction with the splenic vein has been reported in such cases. However, depending on the site of the tumor and other factors, it may be impossible to leave sufficient length of the splenic vein, making anastomosis difficult. We report two patterns of reconstruction with the right gastroepiploic vein during pancreaticoduodenectomy and total pancreatectomy to prevent left-sided portal hypertension.

**Case presentation:**

The first patient was a 79-year-old man who underwent pancreaticoduodenectomy for pancreatic cancer. The root of the splenic vein was infiltrated by the tumor, and we resected this vein at the confluence of the portal vein. Closure of the portal vein was performed without reconstruction of the splenic vein. To prevent left-sided portal hypertension, we anastomosed the right gastroepiploic vein to the middle colic vein. Postoperatively, there was no suggestion of left-sided portal hypertension, such as splenomegaly, varices, and thrombocytosis. The second case was a 63-year-old woman who underwent total pancreatectomy for pancreatic cancer. The splenic vein–superior mesenteric vein confluence was infiltrated by the tumor, and we resected the portal vein, including the confluence. End-to-end anastomosis was performed without reconstruction of the splenic vein. We also divided the left gastric vein, left gastroepiploic vein, right gastroepiploic vein, and right gastric vein, which resulted in a lack of drainage veins from the stomach and severe gastric vein congestion. We anastomosed the right gastroepiploic vein to the left renal vein, which improved the gastric vein congestion. Postoperatively, imaging confirmed short-term patency of the anastomosis site. Although the patient died because of tumor progression 8 months after the surgery, no findings suggested left-sided portal hypertension, such as varices. Reconstruction with the right gastroepiploic vein during pancreaticoduodenectomy and total pancreatectomy is useful to prevent left-sided portal hypertension.

## Background

Pancreaticoduodenectomy (PD) with portal vein (PV) resection is now well accepted as a standard operation for advanced pancreatic head cancer with vascular invasion. Depending on the site of the tumor, the splenic vein (SV) is often divided to achieve margin-negative resection [1]. In addition, when total pancreatectomy (TP) is performed for chronic pancreatitis, tumors of the whole pancreas, and other pathologies, the SV is frequently ligated [2]. However, ligation of the SV can cause left-sided portal hypertension (LSPH) including gastric/splenic venous congestion, which can result in serious intra- or postoperative complications [3].

Several intraoperative strategies have been performed to avoid LSPH. For instance, SV–PV anastomosis [1], SV‐inferior mesenteric vein (IMV) anastomosis [1, 4–6], SV–renal vein anastomosis [1, 3, 7–10], and other anastomoses [1, 11, 12] have been performed. However, owing to the tumor location, it may be impossible to leave sufficient length of the SV; therefore, SV anastomoses may be difficult. Herein, we report our experience with two patterns of reconstruction using the right gastroepiploic vein (RGEV) during PD/TP to prevent LSPH. One patient underwent RGEV–middle colic vein (MCV) anastomosis, and the other underwent RGEV–left renal vein (LRV) anastomosis.

## Case presentation

### Case 1

**Fig. 1:**
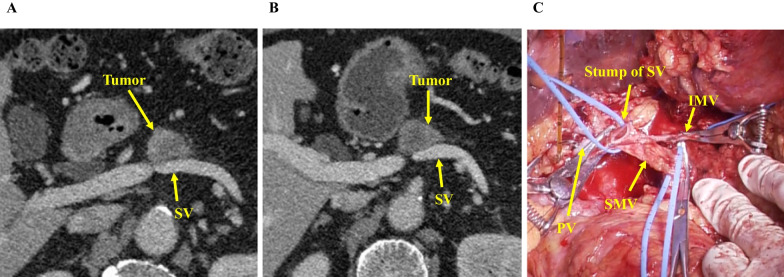
CT before the surgery and operative findings. **A** CT before neoadjuvant chemotherapy showing the tumor near the SV. **B** CT after chemotherapy showing that the tumor is almost the same size as that before chemotherapy and located close to the SV. **C** We resected the SV at the confluence of the PV. *CT* Computed tomography, *SV* Splenic vein, *PV* Portal vein, *SMV* Superior mesenteric vein, *IMV* Inferior mesenteric vein

A 79-year-old man was referred to our hospital because of upper abdominal pain. Ultrasonography revealed a hypoechoic tumor in the pancreatic body, and the endoscopic ultrasonography-guided fine needle aspiration revealed adenocarcinoma. Computed tomography (CT) revealed a mass measuring 22 mm in diameter that was in contact with the SV–superior mesenteric vein (SMV) confluence (Fig. 1A). There were no enlarged lymph nodes and no metastasis. We performed neoadjuvant chemotherapy with two courses of gemcitabine and TS-1. Although CT indicated that the mass measured 22 mm in diameter and was in contact with the SV, we judged that the mass was resectable (Fig. 1B). The preoperative diagnosis was pancreatic cancer, Pb, infiltrative type, TS2 (22 mm), ycT3 (S1, RP1), ycN0, ycM0, and ycStage IIA.

**Fig. 2:**
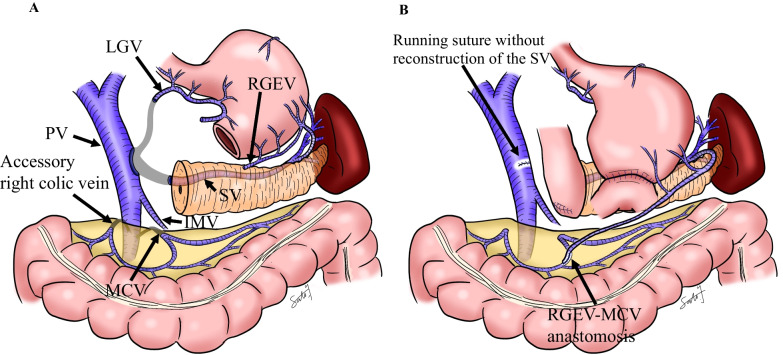
Schema. **A** Schema after vascular resection during pancreaticoduodenectomy. **B** Schema after venous reconstruction. *SV* Splenic vein, *PV* Portal vein, *IMV* Inferior mesenteric vein, *RGEV* Right gastroepiploic vein, *MCV* Middle colic vein, *LGV* Left gastric vein

We performed subtotal stomach-preserving PD-II-A-1 with SV resection. The root of the SV was infiltrated by the tumor, and we resected the SV at the confluence of the PV (Fig. 1C). We used a running suture to close the PV without reconstruction of the SV. The IMV joined the SMV at the caudal side of the SV–SMV confluence, and we preserved the IMV. It was necessary to completely resect the MCV, accessory right colic vein, left gastric vein (LGV), and the gastrocolic trunk. We also divided the RGEV 1 cm on the oral side of the pyloric ring and resected the right gastric vein. The main venous drainage routes from the stomach and the spleen were not retained, and to prevent LSPH, we decided to perform venous reconstruction. Because the site and size of the vessel were suitable, and anastomosis did not interfere with the gastrojejunostomy, we anastomosed the gastric side stump of the RGEV with the colonic side stump of the MCV side-to-side using a running suture with 7–0 Prolene (Ethicon Inc., Somerville, NJ) (Fig. 2A and B). The venous anastomosis was performed by a transplant surgeon.

**Fig. 3:**
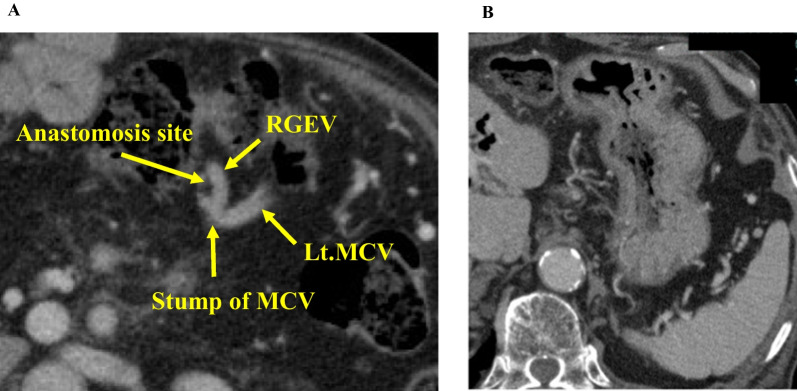
CT after the surgery. **A** CT 2 years after the surgery showing the patency of the RGEV–MCV anastomosis. **B** CT 2 years after the surgery showing the absence of varices around the stomach. *CT* Computed tomography, *RGEV* Right gastroepiploic vein, *MCV* Middle colic vein

There were no major postoperative complications, and the patient was discharged from the hospital on the 21st postoperative day. Adjuvant chemotherapy with S-1 was administered for 6 months, and there was no recurrence for 2 years after the operation. CT confirmed the patency of the anastomosis site (Fig. 3A). There were no findings suggesting LSPH, such as splenomegaly, varices, and thrombocytosis (Fig. 3B).

### Case 2

**Fig. 4:**
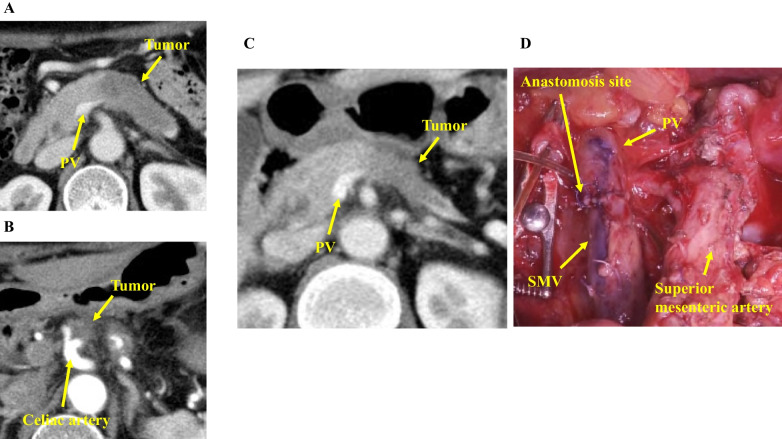
CT before the surgery. **A** CT before neoadjuvant chemotherapy showing the tumor infiltrating the pancreas. **B** On CT before neoadjuvant chemotherapy, the tumor was close to the celiac artery. **C** CT after chemotherapy showing a decrease in the tumor size; however, the tumor is close to the PV. **D** We resected the PV, including the SV–SMV confluence. *CT* Computed tomography, *PV* Portal vein. *SV* Splenic vein, *SMV* Superior mesenteric vein

A 68-year-old woman underwent examination because of worsening diabetes and was diagnosed with pancreatic cancer. CT revealed a tumor extending from the pancreatic head to the pancreatic tail that was near the celiac artery (Fig. 4A and B). The tumor infiltrated the splenic artery and SV, which was obstructed. We administered neoadjuvant chemotherapy with gemcitabine and nab-paclitaxel. On CT 6 months later, although the mass was located along most of the length of the pancreas, the tumor had shrunk locally, and a small distance was observed between the tumor and the celiac artery; therefore, we judged that the mass was resectable (Fig. 4C). Our preoperative diagnosis was pancreatic cancer, Pbht, TS3 (45 mm), ycT3 (S1, RP1, PV1, A1 < Ach, Asp > , PL1), ycN0, ycM0, ycStage IIA, and we performed TP with celiac axis resection.

**Fig. 5:**
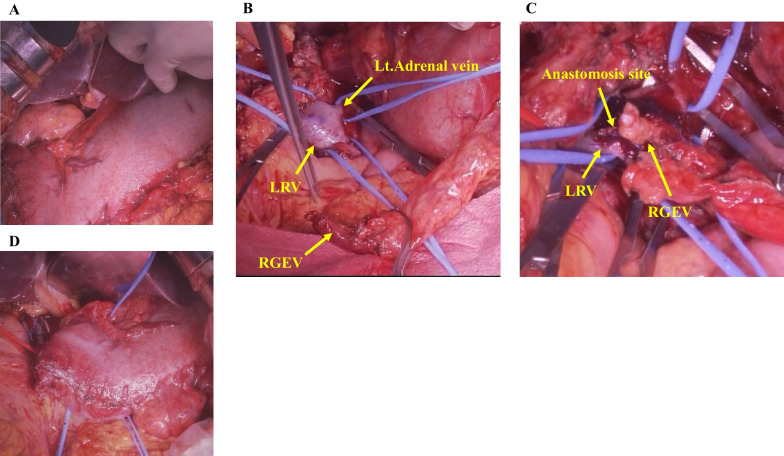
Operative findings. **A** Severe gastric vein congestion was observed during the operation. **B** The RGEV was mobilized by incising the omentum, and the LRV was exposed. **C** We anastomosed the RGEV with the LRV side-to-side using a running suture. **D** The gastric vein congestion improved after venous reconstruction. *RGEV* Right gastroepiploic vein, *LRV* Left renal vein

**Fig. 6:**
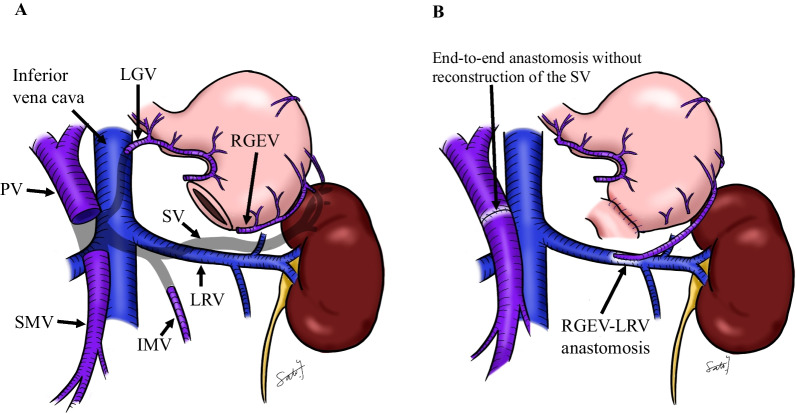
Shema. **A** Schema after vascular resection during total pancreatectomy. **B** Schema after venous reconstruction. *SV* Splenic vein, *PV* Portal vein, *IMV* Inferior mesenteric vein, *RGEV* Right gastroepiploic vein, *LGV* Left gastric vein, *LRV* Left renal vein, *SMV* Superior mesenteric vein

The SV–SMV confluence was infiltrated by the tumor, and we resected the PV with the SV–SMV confluence. End-to-end anastomosis was performed without reconstruction of the SV (Fig. 4D). The IMV was resected at the lower side of the pancreas, and it was necessary to resect the MCV and the LGV. We also divided the RGEV 3 cm on the oral side of the pyloric ring and resected the right gastric vein and left gastroepiploic vein. As a result, there was no drainage vein from the stomach, and severe gastric vein congestion was observed (Fig. 5A). We anastomosed the gastric side stump of the RGEV with the LRV side-to-side using a running suture with 6–0 Prolene (Ethicon Inc.) (Fig. 5B and C), and the gastric vein congestion improved after reconstruction (Fig. 5D). We placed a small number of sutures to fix the anastomotic site in the fatty tissue to prevent rupture of the anastomotic site. The venous anastomosis was performed by a transplant surgeon. The schema after vascular resection during TP and the schema after venous reconstruction are shown in Fig. 6A and B.

**Fig. 7:**
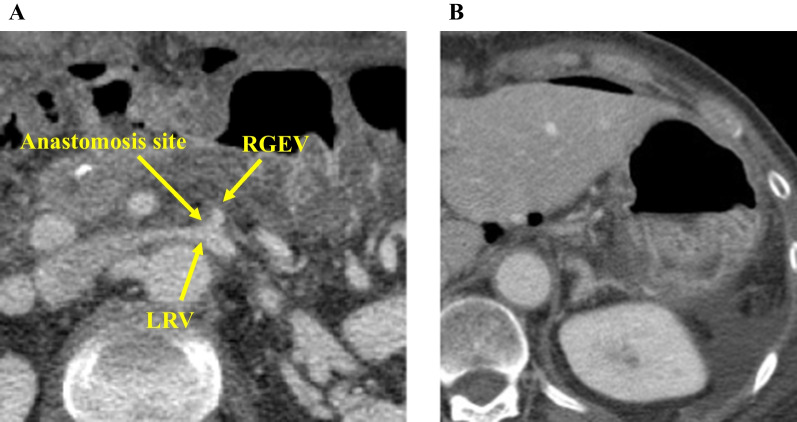
CT after the surgery. **A** CT 6 days after the surgery showing the patency of the RGEV–LRV anastomosis. **B** CT 6 days after the surgery showing the absence of varices around the stomach. *CT* Computed tomography, *RGEV* Right gastroepiploic vein, *LRV* Left renal vein

There were no major postoperative complications, and the patient was discharged from the hospital on the 20th postoperative day. CT 6 days after the operation confirmed the patency of the anastomosis site (Fig. 7A). There were no findings that suggested LSPH, such as varices (Fig. 7B). However, multiple liver metastases were observed 2 months after the operation, and the palliative chemotherapy with FOLFIRINOX was administered. The patient died because of tumor progression 8 months after the surgery.

## Discussion

In this study, we described our experience with two patterns of reconstruction using the RGEV during PD and TP to prevent LSPH including gastric venous congestion. One pattern is RGEV–MCV anastomosis and the other is RGEV–LRV anastomosis. Both patients had short-term shunt patency and showed no signs of LSPH. There were no procedure-related morbidities during the postoperative follow-up.

SV ligation during PD or TP may result in the development of LSPH. LSPH is caused by insufficient splenic/gastric venous drainage and may induce splenomegaly, varices, and severe gastric bleeding/congestion. Variceal bleeding after LSPH is recurrent or massive in some patients, resulting in fatal hypovolemic shock. Splenomegaly causes pancytopenia, resulting in anemia, compromised status, and easy bleeding [13–15]. Regarding TP, Loos et al. [16] reported that the overall 90-day mortality after TP was 4.1%, and 7.4% in patients with gastric vein congestion and 2.8% in those without gastric vein congestion. The authors also reported that half of the patients who died after TP had gastric vein congestion. Mehrabi et al. [17] also described gastric vein congestion after TP, which led to gastric venous infarction and ischemia with subsequent gastric perforation and abdominal sepsis. These complications increase patient morbidity and mortality, and reconstruction of gastric venous drainage is useful to avoid gastric vein congestion. In addition to these postoperative complications, gastric vein congestion can increase difficulty controlling intraoperative hemostasis, which affects the surgery. For these reasons, it is important to prevent LSPH. We have experienced 86 cases of PD with PV resection in the 10 years from 2013 to 2022, of which the SV was sacrificed in 40 cases. LSPH occurred in eight cases, in which perigastric varices, gastric congestion, or splenomegaly occurred, among the cases with SV resection. Although LSPH is a rare complication, its frequency cannot be ignored.

Tanaka et al. [14] reported that the risk of LSPH after PD with portomesentericosplenic confluence resection could be stratified based on the number of preserved critical veins among the LGV, MCV, and superior right colic vein arcade. The authors reported that, in patients who underwent SV ligation during PD in whom none of the three critical veins were preserved and the SV was not reconstructed (*n* = 29), all patients developed LSPH. In patients with only one of the critical veins preserved and no SV reconstruction (*n* = 51), 12 of the 51 (24%) patients developed LSPH. In contrast, no patients with preservation of two or three of the critical veins (*n* = 8) developed LSPH. The authors also reported that in patients with no preserved critical veins who underwent successful SV reconstruction (*n* = 5), LSPH developed in three of the five patients. In addition, in patients with only one preserved critical vein and successful SV reconstruction (*n* = 10), none developed LSPH. Therefore, regarding the indications for venous reconstruction when SV ligation is performed during PD or TP, the number of preserved critical veins is helpful. In our cases, only one critical vein, the right colic vein arcade, was preserved in both case 1 and case 2. In case 2, gastric venous congestion was observed, which is considered a good indication for venous reconstruction. In addition, in case 1, considering the incidence of LSPH in the above study in patients with only one remaining critical vein, and the usefulness of venous reconstruction, we believe that venous reconstruction was appropriate.

Partial gastrectomy is an alternative to venous reconstruction. Nakao et al. [18] reported that distal gastrectomy may be a safe method with which to prevent gastric venous congestion and bleeding when combined with TP. However, extended resection of the stomach with pancreatectomy may lead to functional and structural dysfunction, resulting in worsening of the patient’s nutritional status [2].

Regarding venous reconstruction, there are many reports of the usefulness of reconstruction with anastomosis with the SV, such as SV–SMV anastomosis [1], SV–IMV anastomosis [1, 4–6], SV–renal vein anastomosis [1, 3, 7–10], and other anastomoses [1, 11, 12]. We have experienced a case in which venous reconstruction using the SV was performed during PD with SV ligation. In the case, we performed SV–IMV anastomosis, and there were no findings suggesting LSPH, such as splenomegaly, varices, and thrombocytosis. However, these anastomoses are complicated because the length of the resected SV is long owing to tumor invasion, and it is necessary to separate the SV from the pancreatic parenchyma [15]. In this situation, we suggest performing anastomosis with the RGEV.

To date, there have been few reports of anastomosis using gastric veins (Table 1). In our search of PubMed, we found five cases of reconstruction using a gastric vein during PD or TP [2, 18–20]. In four cases, reconstruction was performed using the LGV [2, 19, 20], and right gastric vein reconstruction was also performed in one of these cases [20]. In the other case, reconstruction was performed with the RGEV, as in our report [18]. In the previous case, emergency anastomosis between the RGEV and the left ovarian vein was performed because severe gastric vein congestion and bleeding occurred during TP. Hemostasis was achieved after the reconstruction.

**Table 1:** Previously reported cases of vascular reconstruction using a gastric vein to prevent left-sided portal hypertension

Author, year	Age (years)	Sex	Diagnosis	Operation	Reconstruction
Sandroussi and McGilvray, 2010 [19]	42	Female	Pancreatic cancer	TP	LGV–IMV
Barbier et al., 2013 [20]	NA	NA	NA	TP	LGV–NA
Barbier et al., 2014 [20]	NA	NA	NA	TP	LGV–NA right gastric vein–NA
Nakao et al., 2017 [18]	64	Female	P-NET	TP	RGEV–left ovarian vein
Kagota et al., 2020 [2]	60	Female	Pancreatic cancer	SSPPD	LGV–SV
Present study, case 1	79	Male	Pancreatic cancer	SSPPD	RGEV–MCV
Present study, case 2	63	Female	Pancreatic cancer	TP	RGEV–LRV

*TP* Total pancreatectomy, *LGV* Left gastric vein, *IMV* Inferior mesenteric vein, *NA* Not available, *P-NET* Pancreatic neuroendocrine tumor, *RGEV* Right gastroepiploic vein, *SV* Splenic vein, *SSPD* Stomach-preserving pancreaticoduodenectomy, *MCV* Middle colic vein, *LRV* Left renal vein

The RGEV is useful for venous reconstruction for the following reasons. First, the RGEV has a certain distance from the SV and PV, which pancreas cancer often invades. In addition, because the RGEV is located in the greater omentum, this vein is mobilized easily by incising the omentum. Furthermore, side-to-side anastomosis for reconstruction is possible because the distance between the anastomosed vessels is short. However, high mobility of the anastomosis site may cause rupture of the anastomosis as a result of body movements. To prevent rupture of the anastomosis, we placed a small number of sutures to fix the anastomosis in the fatty tissue in case 2.

The following points are important during reconstruction with the RGEV. First, because the diameter of the vein is small, a surgical loupe is necessary for the procedure; however, a surgical microscope is unnecessary. In addition, the vessel walls are thin, so care must be taken for not to tear the vessel during anastomosis. Finally, for the venous reconstruction, we chose a large-diameter vein located close enough to be easily anastomosed. During PD, the blood vessels that can be anastomosed are limited because the pancreas overlies the retroperitoneum, and pancreatic-jejunal anastomosis is part of PD. In contrast, during TP, a relatively large number of blood vessels are options, such as the LRV or left gonadal vein, because the anterior layer of Gerota’s fascia is completely exposed, and the vein can be mobilized easily. In case1, because the site and size of the vessel were suitable, and anastomosis did not interfere with the gastrojejunostomy, we anastomosed the gastric side stump of the RGEV with the colonic side stump of the MCV side-to-side. In case 2, we chose the LRV because the anterior layer of Gerota’s fascia is completely exposed, and the LRV, which had a large diameter and was close to the RGEV, could be easily mobilized.

## Conclusions

Reconstruction with the RGEV during PD/TP is useful to prevent LSPH, especially because SV reanastomosis is difficult owing to the site of the tumor.

## Data Availability

Not applicable to this paper as no datasets were generated during this study.

## Abbreviations

PD: Pancreaticoduodenectomy
SV: Splenic vein
TP: Total pancreatectomy
LSPH: Left-sided portal hypertension
PV: Portal vein
IMV: Inferior mesenteric vein
RGEV: Right gastroepiploic vein
MCV: Middle colic vein
LRV: Left renal vein
CT: Computed tomography
SMV: Superior mesenteric vein
LGV: Left gastric vein
